# Recognition of *Candida albicans* and Role of Innate Type 17 Immunity in Oral Candidiasis

**DOI:** 10.3390/microorganisms8091340

**Published:** 2020-09-02

**Authors:** Anna Pavlova, Irshad Sharafutdinov

**Affiliations:** 1 Integrated Immunology Programme, Division of Genetics, Department of Biology, Friedrich Alexander University Erlangen-Nuremberg, 91058 Erlangen, Germany; anna.pavlova@fau.de; 2 Division of Microbiology, Department of Biology, Friedrich Alexander University Erlangen-Nuremberg, 91058 Erlangen, Germany

**Keywords:** *Candida albicans*, innate immunity, IL-17, receptor tyrosine kinase, γδ T cells, natural Th17 cells, iNKT, ILC3

## Abstract

*Candida albicans* is an opportunistic pathogenic fungus considered to be a common member of the human microflora. Similar to some other opportunistic microbes, *C. albicans* can invade and benefit from its host when the immune status of that host is weakened. Most often this happens to immunocompromised individuals, leading to the infection of oral and vaginal mucosae or the systemic spread of the pathogen throughout the entire body. Oropharyngeal candidiasis (OPC) occurs in up to 90 percent of patients with acquired immunodeficiency syndrome (AIDS), making it the most frequent opportunistic infection for this group. Upon first signs of fungal invasion, a range of host signaling activates in order to eliminate the threat. Epithelial and myeloid type cells detect *C. albicans* mainly through receptor tyrosine kinases and pattern-recognition receptors. This review provides an overview of downstream signaling resulting in an adequate immune response through the activation of various transcription factors. The study discusses recent advances in research of the interleukin-17 (IL-17) producing innate cells, including natural T helper 17 (nTh17) cells, γδ T cells, invariant natural killer T (iNKT) cells and type 3 innate lymphoid cells (ILC3) that are involved in response to oral *C. albicans* infections.

## 1. Introduction

The oral environment is characterized by a complex microflora consisting of fungal and bacterial species tightly coexisting with each other [1]. These two major microbial groups can have both synergistic and antagonistic interactions therefore suppression of one group can affect the growth possibilities for another [2]. *Candida albicans* can play a protective function for bacteria as has recently been reported for the dual-species infection *C. albicans* and a periodontal pathogen *Porphyromonas gingivalis* [3]. In contrast, treatment of the denture stomatitis patients with probiotic *Streptococcus salivarius* strain reduced clinical signs and symptoms of infection and the count of *C. albicans*, keeping the effect for at least 30 days [4]. Among all fungal microbes isolated from clinical specimens, the most frequently found species is *C. albicans* [5,6]. This microorganism is a common member of the human microbiota for at least half of the human population [7,8]. *C. albicans* is a highly adaptive microorganism, with the ability to transform between yeast and hyphal forms being one of its major virulence factors [9,10]. When *C. albicans* transforms from yeast to hyphal state, its cell wall undergoes constitutional changes, decreasing the recognition of the pathogen by the host immune system [11,12]. Under certain conditions, *C. albicans* can cause a range of opportunistic diseases, predominantly in immunocompromised patients [13,14]. Classical factors predisposing individuals to *C. albicans* invasion include treatment with broad-spectrum antibiotics, antineoplastic and immunosuppressive treatment, and organ transplantation [15]. In the acquired immunodeficiency syndrome (AIDS) patients, *Candida* was found to be the only fungi isolated from oral mucosal swabs, while in healthy people, mycobiota are more diversified and may include *Cladosporium*, *Aspergillus*, *Fusarium*, *Penicillium* and other genera with *Candida* accounting for around only 20% [16,17]. In general, human immunodeficiency virus (HIV) infected patients have oropharyngeal candidiasis (OPC) as the most common oral manifestation [18,19,20]. Even though much progress has been made over the last two decades in the development of antiretroviral therapy, 38% out of 37.9 million HIV infected patients still have no access to these life-saving medications, making OPC a common disorder for millions of people [21]. When *C. albicans* switches its behavior from benign commensal to aggressive invasion of the epithelial layer, the innate immune system is the first to be activated [22]. The signaling cascade starts from the host–pathogen contact in epithelial cells, following with the interleukin-17 (IL-17)-dependent recruitment of phagocytic cells, such as neutrophils, macrophages, and dendritic cells (DCs) to the site of infection [23,24]. Phagocytes kill fungal cells both intracellularly and extracellularly via oxidative (reactive oxygen species) and nonoxidative mechanisms (hydrolytic enzymes and antimicrobial peptides), which are then followed by adaptive immunity [22,25]. Interestingly, in addition to phagocytosis, neutrophils can constrain the growth of *C. albicans* with neutrophil extracellular traps (NETs) containing calprotectin, an antifungal peptide [26,27]. The main source of IL-17 in humans are T helper 17 (Th17) cells, whereas in mice it is mainly produced by γδT cells and natural Th17 (nTh17) cells [28]. Besides γδT cells and nTh17, there are other IL-17 producing cells, including invariant natural killer T (iNKT) cells and innate lymphoid cells (ILCs), which altogether present type 17 cells [29,30,31]. It is noteworthy that IL-17 dependent immunity in oral candidiasis is considered as a key response to eliminate infection. In vaginal candidiasis, however, its role remains controversial [32,33,34]. In this review, initial pathogen recognition by oral epithelial as well as myeloid cells will be first described. The following role of IL-17 producing innate γδT cells, nTh17, iNKT, and ILC3 cells will be then summarized.

## 2. How Does *C. albicans* Invasion Trigger IL-17 Production?

Organization of the *C. albicans* cell wall is important to understand how this microorganism can be recognized by the host immune system. The cell wall of *C. albicans* consists of two major layers, which define the fungal structure and antigenic properties. The inner β-glucan–chitin skeleton is responsible for the strength and shape of the cell wall. Chitin is located in the inner layer of the cell wall, while β-1,6-glucans are linked to β-1,3-glucans, connecting the inner and outer cell wall [35,36]. The outer layer of *C. albicans* is covered by cell wall proteins which are N- and/or O- glycosylated with mannose-containing carbohydrates (mannans). Outer mannoproteins are cross-linked to the inner β-1,6-glucans through a glycosylphosphatidylinositol (GPI) remnant [37]. Altogether ß-glucans, mannans as well as nucleic acids of *C. albicans*, form a group of so-called pathogen- associated molecular patterns (PAMPs) [38,39]. Identification of nonself molecules via PAMPs can occur by pattern-recognition receptors (PRRs) in both epithelial and myeloid cells. PRRs consist of three major classes: Toll-like receptors (TLRs), C-type lectin receptors (CLRs) and nucleotide-binding oligomerization domain-like (NOD-like) receptors (NLRs) [38,40].

**Figure 1:**
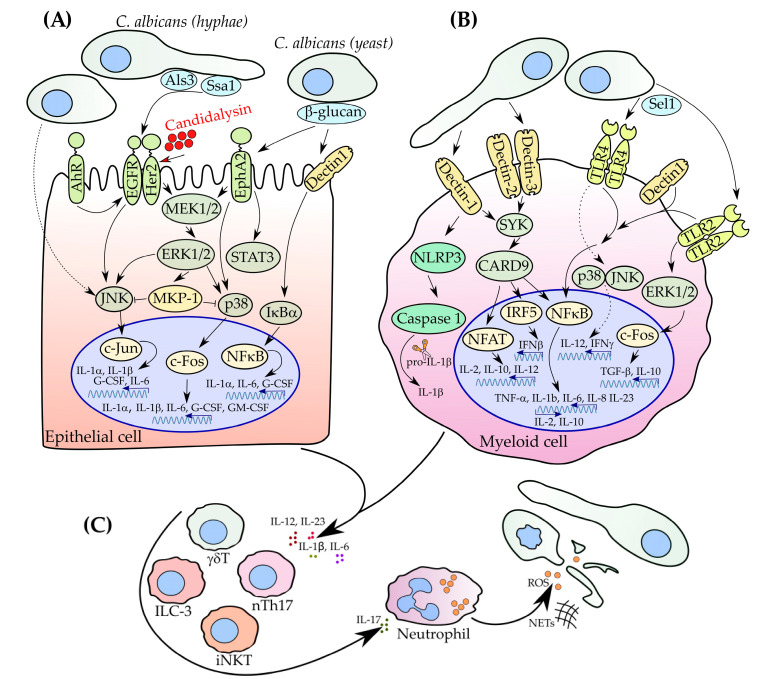
Schematic overview of molecular pathways in response to *C. albicans* which lead to the interleukin-17 (IL-17)-dependent neutrophil recruitment. (**A**) Upon *C. albicans* invasion, epithelial cells recognize fungal pathogen mainly via receptor tyrosine kinases (RTKs) such as epidermal growth factor receptor (EGFR) and human epidermal growth factor receptor 2 (Her2). Phosphorylation events lead to the downstream signaling cascade resulting in the activation of transcription factors and subsequent release of cytokines and chemokines. (**B**) Immune cells of myeloid nature, such as macrophages and dendritic cells (DCs), recognize *C. albicans* mainly via pathogen-associated molecular patterns (PAMPs) recognition by pattern-recognition receptors (PRRs) (key receptors here are dectin-1 and Toll-like receptors 2/4 (TLR2/4)). (**C**) Activated type 17 cells produce IL-17 to recruit neutrophils to the site of pathogen invasion. Neutrophils release reactive oxygen species (ROS) molecules and neutrophil extracellular traps (NETs) and provide the host cells protection, especially during the first hours of infection.

However, the molecular basis of pathogen recognition and signal transduction upon *C. albicans* infection differs in epithelial and myeloid cells. In general, the response to *C. albicans* begins with the activation of (1) receptor tyrosine kinases (RTKs), mainly in epithelia, and (2) PRRs predominantly in myeloid cells (Figure 1). Activated receptors subsequently trigger molecular signaling to transcription factors resulting in cytokines/chemokines release. In addition, activated receptors can initiate secretion of antimicrobial peptides, such as cathelicidin LL-37, histatins, and defensins, suppressing pathogen growth [38,41].

### 2.1. Role of Myeloid Cells in Pathogen Recognition

Myeloid cells, such as macrophages and DCs, mainly respond to *C. albicans* through PRRs recognition of respective PAMPs. CLR family members dectin-1 and dectin-2/3 participate in recognition of *C. albicans* by binding to fungal β-glucans and α-mannans, respectively [42,43]. Dectin 1 is mainly expressed on monocytes and macrophages, while Dectin 2 is expressed on dendritic cells (DCs), macrophages, and neutrophils [44,45]. After binding to the ligands, dectins recruit the spleen tyrosine kinase (SYK) via their tyrosine-based motifs [46,47]. Caspase recruitment domain 9 (CARD9) protein is a key transducer of Dectin-1 signaling for activation of myeloid cells, cytokine production, and innate antifungal immunity [42,45,48]. The downstream signaling leads to activation of the nuclear factor kappa-light-chain-enhancer of activated B cells (NF-κB), whereby coordinating the induction of tumor necrosis factor (TNF), interleukin (IL)-1β, IL-6, and IL-23 in DCs and macrophages [43,47,49]. Alternatively, downstream signaling from dectin-1 to SYK and CARD9 can activate the interferon response factor 5 (IRF5), resulting in interferon β production. Dectin-1 was also shown to activate the nuclear factor of activated T-cells (NFAT) transcription factor, resulting in the production of IL-2, IL-10, and IL-12 p70 by both macrophages and dendritic cells [50,51]. In mucosal candidiasis, dectin-1 can induce a robust production of the p19 chain of IL-23 and a low induction of the p35 chain of IL-12. However, this was shown to be dependent on host genetic background [52]. IL-23p19(-/-) mice with impaired Th17 response developed severe OPC and diminished neutrophil recruitment, while Th1-deficient IL-12p35(-/-) mice countered infection, assuming a dispensable role of Th1 response during OPC [24]. Since IL-12 and IL-23 heterodimers share a common p40 chain, dectin 1-dependent p19 production can presumably define Th17 differentiation. Interestingly, *C. albicans* can escape from dectin-1 recognition via masking β-glucans with cell wall mannoproteins, which causes the delay of fungal uptake by phagocytes, allowing hyphal extension [12]. This mechanism is also well established in another fungal pathogen, *Aspergillus fumigatus*, which also displays stage-specific β-glucan exposure to dectin-1 [53]. Furthermore, in response to *C. albicans* both dectins 1 and 2 were shown to activate host cytosolic phospholipase A2 (cPLA_2_α), following with the production of arachidonic acid metabolites, such as prostaglandins [54,55]. Prostaglandins are potent lipid molecules playing a pivotal role in the modulation of immunity [56,57]. Intriguingly, *C. albicans* is able to produce prostaglandins itself *de novo* or via conversion of exogenous arachidonic acid, thereby amplifying downstream signaling [58,59]. It was shown that fungal prostaglandins can inhibit TNF-α and induce IL-10 production, which can enhance fungal-cell adhesion, germ-tube development, and biofilm formation by *C. albicans* [58,60,61,62]. Other important CLRs recognizing fungal mannans include mannose receptor, DC-specific ICAM3-grabbing non-integrin (DC-SIGN), and macrophage-inducible C-type lectin (MINCLE) [63]. Altogether, CLRs compose a major group of PRRs providing a strong proinflammatory response, which provides initial host protection. On the other hand, TLR2 and TLR4 can recognize fungal O-linked mannoproteins and phospholipomannans, respectively. This leads to the activation of NF-κB and mitogen-activated protein kinases (MAPKs), such as c-Jun N-terminal kinase (JNK), extracellular signal-regulated kinase (ERK) and MAPK p38 (p38), which induce a strong proinflammatory cytokine response [64,65,66]. As it has been shown by in vitro studies, the production of proinflammatory cytokines in response to yeast and hyphal *C. albicans* in macrophages is likely dependent on TLR2 signaling, while TLR4 seems to have a minor role in recognition of this pathogen [65,67]. However, other researchers have demonstrated that both TLR2 and TLR4 are equally important in mediating immune response to *C. albicans*, presenting different points of view in this regard [68,69,70]. Recently, *C. albicans* has been shown to upregulate expression of its small secreted cysteine-rich protein Sel1, and both TLR2 and TLR4 were required for its recognition [69]. In addition, NOD-, LRR- and pyrin domain-containing 3 (NLRP3) can recognize fungal hyphae (but not yeasts) leading to caspase-1 mediated mature IL-1β production [71,72]. Interestingly, *C. albicans* peptide toxin candidalysin was found to trigger both NLRP3 inflammasome response and inflammasome-independent cytolysis of macrophages and dendritic cells [73,74]. Complement system and pattern-recognition molecules (PRMs) also play important roles in innate immune defense against *C. albicans* [75,76]. All main pathways of the complement system, including classical, lectin, and alternative pathways, have been reported to be activated in response to this microbe [76]. C3 fragments opsonize *C. albicans* and enhance the uptake of the pathogen by complement receptor-bearing phagocytes via CR3. Among the PRMs, members of the pentraxin superfamily have been shown to be implicated in anti-fungal immunity [77]. Pentraxin 3 (PTX3) belongs to the long pentraxins and can be secreted by monocytes, macrophages, and dendritic cells in response to TNF and IL-1 signaling [78,79]. PTX3 was demonstrated to form complexes with MBL or ficolin-2 to promote the deposition of complement on surfaces of *C. albicans* and *A. fumigatus*, respectively, resulting in their phagocytosis [80,81]. In addition, the retinoic-acid-inducible gene I (RIG-I) like receptor was also shown to recognize *C. albicans*, but its role remains unclear [82]. However, the mice deficient in the major PRRs TLR2 or dectin-1 were not susceptible to OPC, assuming these signaling pathways to be dispensable for *C. albicans* elimination [83,84]. Hence, other mechanisms involved in fungal recognition by epithelial cells seem to be more important and are discussed in further detail below.

### 2.2. Recognition of C. albicans by Epithelial Cells

In oral epithelial cells, *C. albicans* has been shown to interact with host epidermal growth factor receptor (EGFR) and human epidermal growth factor receptor 2 (Her2) to induce NF-κB pathway activation, especially during the first two hours post- infection (hpi) [85,86]. Major fungal invasins Als3 and Ssa1 can interact with host RTKs, resulting in epithelial cell endocytosis of *C. albicans* [86]. In this process, the toxin called candidalysin plays a major role in the activation of immune response through EGFR tyrosine phosphorylation at Y-845 and Y-1068, [84,87]. Aryl hydrocarbon receptor (AhR) was shown to mediate EGFR phosphorylation at Y-1068 via activation of Src family kinases [88]. In its turn, this results in the activation of ERK1/2, JNK and p38 proteins only 15 min post- infection, indicating early activation of epithelial cell response [85]. Notably, at 2 hpi ERK1/2 phosphorylated MAPK phosphatase (MKP1) resulting in downregulation of p38 and JNK, which prevented excessive cytokine production (in particular granulocyte colony stimulating factor (G-CSF) and granulocyte/macrophage colony stimulating factor (GM-CSF)) [85,89]. Another transcription factor activated upon *C. albicans* invasion was found to be a proto-oncogene c-Fos which, in contrast to JNK and p38, requires hypha formation by the pathogen [85,90]. Experiments with specific inhibitors revealed that in response to *C. albicans*, NF-κB promotes transcription of IL-1α, IL-6, and G-CSF, while JNK stimulates production of IL-1α, IL-1β, G-CSF, and to a lesser extent, IL-6. On the other hand, inhibition of the p38/c-Fos axis resulted in significant reduction of IL-1α, IL-1β, IL-6, G-CSF and GM-CSF levels [85]. Therefore, c-Fos appears to be a major regulator of inflammatory response in oral epithelial cells, in particular upon *C. albicans* invasion and candidalysin release. Another RTK ephrin type-A receptor 2 (EphA2) has been recently shown to interact with β-glucans of yeast-phase *C. albicans,* which results in downstream phosphorylation of MAPK/ERK kinase 1/2 (MEK1/2), p38-mediated activation of c-Fos and signal transducer and activator of transcription 3 (STAT3) [91]. Inhibition of EphA2 signaling was observed to block *C. albicans*-induced EGFR phosphorylation, while inhibition of EGFR decreased EphA2 phosphorylation, suggesting that a reciprocal interaction between EphA2 and EGFR governs the endocytosis of *C. albicans* [91]. Furthermore, increase in fungal load (high multiplicity of infection) resulted in increased phosphorylation of EphA2 and MEK1/2 following with enhanced secretion of IL-8 and human β-defensin 2. Inhibition of EphA2 in the wild type mice dramatically reduced mRNA levels of *Il17a* (350-fold), *Il22* (1000-fold), S100a8 (nine-fold), and Defb3 (sevenfold), assuming a major role of EphA2 in the host inflammatory response to *C. albicans* [91]. Besides, PRR mediated response to *C. albicans* infection has also been shown to occur in oral epithelial cells. TLR4 expression in epithelial cells required addition of polymorphonuclear leukocytes (PMLs) and increased in a time-dependent manner with 100-fold upregulation after 24 h [92]. C-type lectin dectin-1 was shown to induce NF-κB signaling in oral epithelial cells, though it had a limited role in the epithelial cell response to *C. albicans* [91]. Both TLR4 and dectin-1 were shown to play a modest role in IL-8 signaling, known to be important in the recruitment of PMLs such as macrophages and neutrophils.

## 3. IL-17 Orchestrates Innate and Adaptive Immunity

The IL-17 cytokine family comprises six members that participate in both acute and chronic inflammatory responses. IL-17A cytokine was discovered first, and other members of this family (IL-17B, IL-17C, IL-17D, IL-17E, and IL-17F) have been identified later based on amino acid sequence homology [93,94,95,96,97]. The most extensively studied cytokine of this family is IL-17A—a pro-inflammatory cytokine that plays a crucial role in host defense against bacterial and fungal infections. Apparently, IL-17 signaling is essential for host protection against *C. albicans* infection, particularly in the oral environment [30]. For instance, mice lacking the IL-17 receptor or its key downstream signaling adaptor Act1 were highly susceptible to OPC [98]. Furthermore, patients with inherited mutations in the IL-17 receptor signaling or with hyper-IgE syndrome were susceptible to chronic mucocutaneous candidiasis, signifying a role of the IL-17 immunity against *C. albicans* [99]. Potential antifungal activity of IL-17 involves regulation of the expression of antimicrobial peptides and histatins, as well as recruitment and activation of neutrophils [100,101]. Although, neutrophil recruitment and activity were not impaired in IL-17RA- and IL-17RC-deficient mice or in mice with depletion of IL-17A and IL-17F cytokines [102]. The major source of IL-17 is classically considered to be Th17 effector cells; however, it can also be produced by other cell types, the most prominent of which are various innate cells collectively called Type 17 cells [29]. Type 17 cells can provide quick production (within hours) of IL-17 in response to pathogens or tissue injury. This group includes certain nTh17 cells, γδ T cells, iNKT and an innate lymphoid cell (ILC) population known as ILC3 [31]. IL-17-secreting cells represent a small cell population in healthy oral tissues in mice and humans [30]. In healthy human gingiva Th17 cells constitute most IL-17 secreting cells, while in mice, the predominant IL-17-secreting cells are γδ T cells, followed by nTh17 cells and ILC3s, though their contribution is controversial [28]. Thereby, a range of innate cells provides initial IL-17 production, which in turn orchestrates various immune events to provide protection against *C. albicans*.

### 3.1. nTh17 Cells

Natural IL-17-producing T cells were first described by Marks et al. in 2009 [103]. These cells develop in the thymus and enter the bloodstream as mature cells expressing IL-23R, CD4, CCR6, α4β1 (VLA4, CD49d/CD29) and TCR-αβ surface receptors as well as retinoic acid-related orphan receptor gamma t (RORγt) transcription factor [103,104,105]. nTh17 cells are intrathymically selected based on agonist stimulation by self-antigen as was shown in AND × PCC double-transgenic mice (B10.BR mice bearing the AND TCR transgene specific for a peptide of pigeon cytochrome c (PCC) crossed with mice expressing pigeon cytochrome c under control of an MHC class I promoter) [103]. Intact medullary epithelial compartments and MHC class II expression by thymic stroma are essential for nTh17 cells development, while expression of the autoimmune regulator (Aire), CD80/86 and ICOS-ligand co-stimulatory molecules regulates nTh17 development. Interestingly, in comparison with the reported role of the inducible nitric oxide synthase (iNOS) in the negative regulation of conventional Th17, thymic nTh17 cells do not require iNOS activity [106]. In AND × PCC double-transgenic animals, nTh17 cells were shown to mainly localize in peripheral organs including lamina propria, liver, lung and Peyer’s patches [103]. However, another study indicated that nTh17 cells were abundant in lymph nodes, but not in lamina propria [107].

Even though the sequencing of the nTh17 TCR repertoire revealed a high degree of diversity, nTh17 cells possess innate-like features [31]. First, nTh17 cells, similar to innate type γδ T cells, secrete IL-17 as a major proinflammatory mediator and are capable of modulating peripheral inflammatory responses. Then, same as other innate-like T cells (for instance iNKT), nTh17 cells express high levels of the transcriptional repressor promyelocytic leukemia zinc finger (PLZF). Along with PLZF protein, RORγt serves as a characteristic marker for the identification of nTh17 cells in the thymus and periphery [107]. Although nTh17 cells in some aspects are similar to inducible Th17 (iTh17), for instance in expression of RORγt and production of IL-17 and IL-22, nTh17 are suggested to differ from peripherally induced Th17 via their innate-like ability to produce IL-17A and IL-22 after TCR-independent TLR-driven cytokine stimulation driven by TLR activation (i.e., IL-1β and IL-23) [31]. This was shown by the administration of imiquimod (IMQ), a TLR7 ligand, which increased the in vivo production of IL-17 in nTh17 cells. Furthermore, in IMQ-treated mice, these cells represent one of the major IL-17–producing T cell subsets present in draining lymph nodes [107]. Unlike iTh17 cells, nTh17 cells did not require IL-6 signaling for their development [105,107], although the opposite assumption was shown first [103]. Little is known about the role of nTh17 cells in infection. Nonetheless, it was demonstrated that lung epithelial cells (LEC) regulate innate antifungal immunity against *Blastomyces dermatitidis* by expanding the numbers of IL-17A- and GM-CSF-producing innate lymphocytes, particularly nTh17 cells in mice. LECs are assumed to regulate the numbers of nTh17 cells via the production of chemokines such as CCL20, whereas this process depends on IL-1α signaling [108]. Conventional Th17 cells were shown to be dispensable for *C. albicans* elimination in the OPC model since IL-6−/− mice were capable of clearing the pathogen from the oral cavity. After 48 hpi in the oral mucosa, *C. albicans* increased the number of nTh17 cells in the tongue approximately twofold [31]. Conversely, nTh17 cells were not detected in tongues of *C. albicans*–infected Rag1−/−, IL-7Rα−/− and in germ-free mice, suggesting that commensal microflora are needed for their development or recruitment to the oral mucosa. Remarkably, there was no expansion of nTh17 cells upon exposure to *Candida glabrata,* showing that different species belonging to the same genus may have distinct immune responses, possibly due to redundancy of Type 17 cells. The authors suggested that nTh17 cells mediating the innate response to OPC may explain the high susceptibility of AIDS patients to this disease, since depletion of CD4+ cells by HIV would likely affect nTh17 cells as well [31].

### 3.2. γδ T Cells

γδT cells are thymus-derived T cells expressing heterodimeric T-cell receptors (TCR) composed of (TCR)γ/TCRδ chains on their cell surface [109,110,111]. γδT cells consist of various subsets which are localized in different anatomical locations and provide first line defense against viral, bacterial and fungal pathogens [112]. For instance, gingival γδT cells of adult mice are mainly composed of Vγ6+ (~60%) and Vγ1+ (~20%), while other subsets Vγ4+ (~10%), Vγ5+ (~5%) and Vγ7+ (~2%) are less enriched in this area [113]. They reside in the basal layer of the oral epithelium and in the junctional epithelium close to the dental biofilm, where they can reach the suprabasal layers [114]. It was demonstrated that during *C. albicans* infection in HIV patients the number of Vδ1 T cells was significantly higher when compared to HIV-patients with no infection. Moreover, *C. albicans* was shown to drive proliferation and IL-17 production by human Vδ1 T cells in vitro [115]. Although some Vδ1 T cells can produce IL-17 upon direct contact with the fungus, a more significant amount of IL-17 production requires the DC-dependent proliferation of Vδ1 T cells [116]. IL-17 production by Vδ1 T cells requires IL-1β, IL-6 and IL-23 secretion by DCs induced by *C. albicans*. However, addition of these cytokines in the absence of DCs was not sufficient to induce Vδ1 T cell proliferation and respective IL-17 secretion. Therefore, direct contact with DCs is required for proliferation of Vδ1 T cells. These results suggest that Vδ1 T cells are likely to be important mediators of immunity against candidiasis, particularly in the HIV infected patients which have much less CD4+ Th17 cells [116]. IL-17 expression by tongue-resident γδT cells in mice is considered to be mediated by Vγ6 cells, which are the main subset of γδT cells in the tongue as well [117,118]. They play an important role in OPC in mice, although TCR-δ−/− mice are mainly resistant to oral candidiasis [31]. γδ T cells can produce high amounts of IL-17 on a per-cell basis [119,120], and thus are potentially an important source of oral IL-17, which plays a crucial role in OPC [30,114]. Interestingly, the development of uterine γδ T cells in mice was independent from microflora, while Vγ6+ γδT cells in the gingiva were shown to be dependent [114,121]. Notably, this salient feature of gingival γδT cells appeared to have a subset-dependency since Vγ4+ γδT cells were unaffected. Therefore, γδT cells activity depends on many factors, such as host and tissue specificity, and immune environment, which implicates complex interplay between *C. albicans* and its host. For instance, deficiency of Vγ6+ γδ T cells was shown to impair recruitment of neutrophils in mice reproductive tract, which severely impaired resistance to *C. albicans* [121]. Kidney-resident γδT cells with a 6- to 7-fold increase were shown to be the primary source of IL-17 during disseminated *C. albicans* infection [122]. Upon infection with *C. albicans,* dermal γδ T cells produce IL-17A to provide resistance to cutaneous candidiasis, and this requires IL-23 production by dermal DCs [123,124]. By summarizing these findings, γδ T cells comprise diverse subsets present in different tissues and play a vital role in IL-17 signaling.

### 3.3. iNKT

iNKT cells are innate-like T lymphocytes which recognize endogenous and foreign lipid antigens presented in the nonpolymorphic MHC-I-like molecule CD1d [125,126]. iNKTs are self-reactive cells expressing a semi-invariant TCR with a canonical TCRα and limited set of TCRVβ chains. These cells play an important role in early immune response against different pathogens and they respond rapidly upon primary TCR stimulation by releasing large quantities of cytokines, such as interferon gamma (IFN-γ), TNF, IL-2, IL-3, IL-4, IL-5, IL-9, IL-10, IL-13, IL-17, IL-21, and GM-CSF [127,128]. In mice, it has been established that iNKT cells can be subdivided into three heterogeneous populations, which resemble subsets of CD4^+^ T cells and innate ILCs [129,130]. For example, iNKT1 cells are similar to Th1 T cells and ILC1s in the expression of transcription factor T-bet and secretion of IFNγ upon activation. iNKT2 cells secrete cytokines that include IL-4 and IL-13, and therefore resemble Th2 cells, whereas iNKT17 cells are similar to Th17 cells with regard to their cytokine secretion profile. Moreover, iNKT cell subsets express different integrins, as well as cytokine receptors, which allows them to populate different tissues after completing the development in the thymus [130,131]. Human iNKT cells have not been studied to an equal extent as murine iNKT cells. However, it was demonstrated that double-negative iNKT cells (CD4^−^ and CD8^−^) in human blood and those that express CD8 (either CD8αα or CD8αβ) are different from CD4^+^ iNKT cells [127,132]. The double-negative and CD8^+^ iNKT cells found in humans were described to resemble mouse iNKT1 cells, showing increased IFNγ secretion and cytotoxic function when activated [127,133]. Recently Erkers et al. suggested that iNKT cells are better described as T-cell–like and NK-like [134]. Killer cell lectin-like receptor subfamily G member 1 (KLRG1) and CD94 markers were used to define iNKT subsets. KLRG1 was expressed in both CD4^+^ and CD4^−^ cells and was associated with the production of Th1 cytokines, while CD4^+^ cells were also able to produce IL-4. CD94 was expressed only by CD4^−^ cells which did not express any IL-4 and displayed elevated cytotoxicity. In addition, human iNKT cells have also been recorded as capable of producing IL-17 in a pro-inflammatory environment [134].

Little is known about the role of iNKT cells in fungal infections. iNKT cells have been shown to be activated by the fungus *Aspergillus fumigatus* both in vitro in the presence of antigen-presenting cells and in vivo after fungal infection [135]. Furthermore, CD1d−/− mice were impaired in their ability to rapidly control *A. fumigatus* infection. Interestingly, similar to *A. fumigatus*, *Histoplasma capsulatum* and *Alternaria alternata*, *C. albicans* activates iNKT cells via fungal β1,3-glucan recognition by Dectin-1 and subsequent IL-12 secretion by activated CD1d-expressing antigen-presenting cells in vitro [135]. In another study, it was shown that cholesteryl 6′-O-acyl α-mannoside, found in *C. albicans* induced activation of mouse and human iNKT cells, dependent on CD1d [136]. However, intravenous *C. albicans* infection of J alpha 18KO mice which specifically lack iNKT cells, has shown that iNKT cells play a minor role in controlling systemic *C. albicans* infections since survival, fungal burden, and production of inflammatory cytokines in several organs did not significantly change during infection [137]. In addition, CD1d−/− mice have also exhibited resistance to oral candidiasis, confirming the marginal role of iNKT cells in *C. albicans* infection [31].

### 3.4. ILC3

ILCs resemble T lymphocytes, but lack adaptive antigen receptors generated by the VDJ recombination. ILC1s, ILC2s and ILC3s are functionally similar to CD4+ T helper cell subsets – Th1, Th2 and Th17 cells, respectively [138,139]. Since ILC3 shares functional similarities with the Th17 subset, it was suggested that ILC3 can be a source of IL-17A during the early stages of OPC, which was confirmed in experimentally infected mice [140]. However, another group reported that IL-17 is primarily expressed during OPC by nTh17 and γδ T cells, but not by ILCs [31]. Perhaps the conflicting results were due to the indirect measurements of IL-17A (*Il17a* and *Il17f* transcripts in crude tongue extracts or IL-17A promoter activity in *Il17a*-eYFP fate reporter mice) production during infection [140]. Interestingly, eYFP-expressing ILC3 cells were not detected in both *C. albicans*–infected and sham kidneys [122]. However, later it was demonstrated by direct visualization of IL-17A and IL-17F cytokines in the infected tongue that three separate and complementary IL-17-producing cell types exist, namely nTh17s, γδ T cells and ILC3s [141]. Thus, these three subsets can compensate for each other’s function. For instance, lack of nTh17 or γδ T cells does not affect fungal control and only deletion of all three subsets resembles the high susceptibility of IL-17RA or IL-17RC-deficient mice to OPC, underlining the robustness of the IL-17 response to the fungus [24].

## 4. Outlook and Perspectives

Persisting *C. albicans* cells challenge oral tissue by stimulation of host intracellular signaling, which in response aims to eradicate exogenous pathogens. Activated transcription factors provide initial recruitment of innate cells, including nTh17, γδT cells, iNKTs and ILC3 cells, aimed to produce IL-17. This important cytokine in OPC initiates and multiplies neutrophil-mediated fungi elimination. However, the role of type 17 cells is still not clear in response to human OPC since the main protection is provided via conventional Th17 cells. These cells may play an important role in protection against *C. albicans* in individuals with CD4^+^ T cell deficiency, for instance in HIV-positive patients. Therefore, better understanding of biology of IL-17 producing cells and their role in human candidiasis may help to develop new approaches for treatment methods. In addition, increased prostaglandin levels during fungal infections can exacerbate fungal colonization and trigger chronic infection [58]. Thus, different enzymes of arachidonic acid pathways, for instance cPLA2α, can serve as a target for the development of new therapeutic strategies against *C. albicans* [62]. Altogether, this may constitute the object of future studies.

In addition, *C. albicans* can escape from the host’s defense mechanisms in multiple ways. Besides genetically determined resistance, *C. albicans* can form rigid biofilms where cells become phenotypically resistant to antimicrobials and the immune system [142,143,144,145]. It appears that we still have a long way to go to understand how the immune system overcomes biofilm-associated resistance. Nevertheless, many studies have already resolved the problem in the context of pathogen recognition. RTK signaling is widely used by microbial pathogens to invade epithelial host cells and hijack their signaling for their own benefits [146,147]. Diverse pathogens such as viruses, bacteria, fungi and protozoa share similar signaling pathways to initiate host cell entry, and RTKs play a central role to initiate this process [147,148,149,150,151]. In this respect many efforts have been made to define Her2, EGFR and EphA2 as growth factor receptors involved in *C. albicans* internalization [86,87,91]. For instance, dysregulation of EGFR in gastric epithelial cells by another pathogen *Helicobacter pylori* has been shown to lead to gastric disorders, such as peptic ulceration and neoplastic transformation [152,153,154]. Association of *C. albicans* with cancer progression is less established; however, analysis of nationwide medical registries in Denmark and Taiwan have reported increased cancer risks upon *C. albicans* infection [155,156,157,158]. Inflammation triggered by *C. albicans*, in particular IL-17-driven, indeed may contribute to the tumorigenesis, as it has been shown for autoimmune disorders [159,160,161,162]. However, the role for innate and adaptive arms of immunity in *C. albicans*-driven tumorigenesis remains widely obscured and further research could elucidate this important question.
